# Gene Perturbation Atlas (GPA): a single-gene perturbation repository for characterizing functional mechanisms of coding and non-coding genes

**DOI:** 10.1038/srep10889

**Published:** 2015-06-03

**Authors:** Yun Xiao, Yonghui Gong, Yanling Lv, Yujia Lan, Jing Hu, Feng Li, Jinyuan Xu, Jing Bai, Yulan Deng, Ling Liu, Guanxiong Zhang, Fulong Yu, Xia Li

**Affiliations:** 1 College of Bioinformatics Science and Technology, Harbin Medical University, Harbin, Heilongjiang 150086, China; 2 Key Laboratory of Cardiovascular Medicine Research, Harbin Medical University, Ministry of Education.

## Abstract

Genome-wide transcriptome profiling after gene perturbation is a powerful means of elucidating gene functional mechanisms in diverse contexts. The comprehensive collection and analysis of the resulting transcriptome profiles would help to systematically characterize context-dependent gene functional mechanisms and conduct experiments in biomedical research. To this end, we collected and curated over 3000 transcriptome profiles in human and mouse from diverse gene perturbation experiments, which involved 1585 different perturbed genes (microRNAs, lncRNAs and protein-coding genes) across 1170 different cell lines/tissues. For each profile, we identified differential genes and their associated functions and pathways, constructed perturbation networks, predicted transcription regulation and cancer/drug associations, and assessed cooperative perturbed genes. Based on these transcriptome analyses, the Gene Perturbation Atlas (GPA) can be used to detect (i) novel or cell-specific functions and pathways affected by perturbed genes, (ii) protein interactions and regulatory cascades affected by perturbed genes, and (iii) perturbed gene-mediated cooperative effects. The GPA is a user-friendly database to support the rapid searching and exploration of gene perturbations. Particularly, we visualized functional effects of perturbed genes from multiple perspectives. In summary, the GPA is a valuable resource for characterizing gene functions and regulatory mechanisms after single-gene perturbations. The GPA is freely accessible at http://biocc.hrbmu.edu.cn/GPA/.

Gene perturbations by knockout, RNA interference (RNAi) or overexpression have been widely used to elucidate gene functions, considerably impacting many areas of biological and medical research over the past decade^1^,^2^. Huge numbers of gene perturbation screens have been performed in many model organisms and in humans. In general, these screens focus on detecting molecules associated with specific biological phenotypes, such as cell morphology, viability, migration and growth rates^3^. The recent development of high-throughput screening techniques further facilitates the comprehensive identification of important genes involved in phenotypes of interest. However, it is difficult to directly characterize the molecular mechanisms of perturbed genes and depict how perturbed genes contribute to specific phenotype changes, such as via interactions with other key genes or inducing the dysfunction of specific biological processes or pathways^4^. Notably, many studies have performed transcriptome analysis of expression profiles measured on microarrays after gene perturbations. For example, Boumahdi *et al.* uncovered a gene network regulated by SOX2 by analyzing the transcriptome profile of SOX2 deletion in squamous-cell carcinoma^5^. Through analyzing the transcriptome profiles of 147 large intergenic non-coding RNA (lincRNA) knockdowns, Guttman *et al.* revealed that lincRNAs mainly regulated global gene expression in trans, maintained the pluripotency and repressed the differentiation of embryonic stem cells^6^. These expression profiles reveal global gene expression changes caused by perturbed genes and can be used to infer their context-dependent biological functions, cellular pathways and regulatory cascades (interacting genes or upstream transcription factors). Thus, it is valuable to identify changes of the functions, pathways and regulatory cascades through gene perturbation, which provide a unique view of the molecular mechanisms of perturbed genes.

Currently, there are many databases serving gene perturbation experiments. Some of these databases provide experimentally validated perturbation reagents (e.g., siRNAs), perturbed model organisms (e.g., knockout mouse) or experimental protocols, such as DEQOR^7^, E-RNAi^8^, IKMC^9^ and ZFIN^10^. Others mainly collect phenotype images or descriptions of gene perturbations, such as GenomeRNAi^11^, IMPC^12^, MPD^13^. To our knowledge, there is no specific database designed to store gene expression profiles produced by gene perturbations and perform corresponding transcriptome analysis, although the transcriptome profiles of gene perturbations are being rapidly accumulated. Thus, the development of such a database will greatly promote the discovery of gene function and regulatory mechanism, facilitating biological and medical research by experimental scientists.

In this study, we collected and analyzed a large number of transcriptome profiles of single-gene perturbations, including protein-coding genes, microRNAs and long non-coding RNAs (lncRNAs), in human and mouse. Integrating these profiles and corresponding transcriptome analysis results, we developed a user-friendly database called the Gene Perturbation Atlas (GPA) with several web tools to support rapid searching, exploration and visualization of the gene perturbations. The GPA provides considerable resources, helping biologists to systematically characterize context-dependent gene functions and regulatory mechanisms and providing references for biomedical gene perturbation experiments conducted by experimental scientists.

## Results

**Figure 1:**
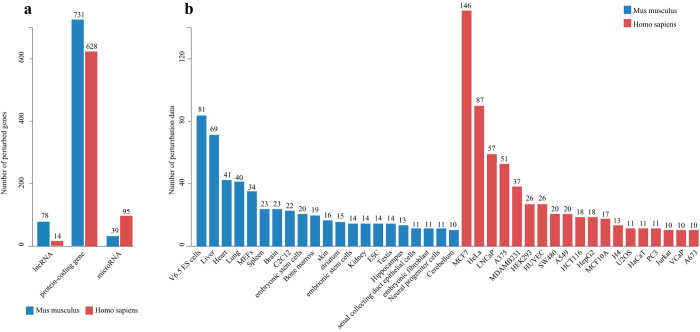
Data statistics of the GPA. **(a)** The number of perturbed lncRNAs, protein-coding genes and microRNAs in human and mouse. **(b)** The number of perturbation datasets involved in primary cell lines or tissues.

**Figure 2:**
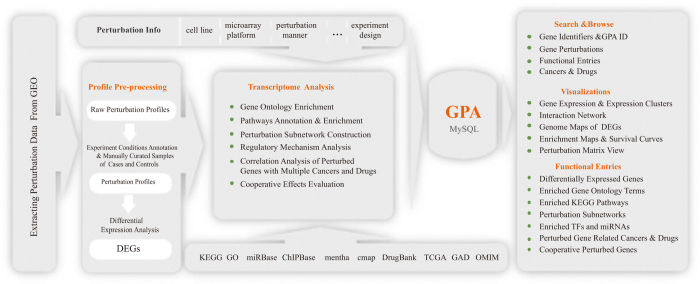
Schematic illustration of the architecture of the GPA.

We manually collected and curated 3072 transcriptome profiles of single-gene perturbations measured on microarrays in human and mouse from Gene Expression Omnibus (GEO). These profiles refer to 1585 different perturbed genes, including 628 protein-coding genes, 95 microRNAs and 14 lncRNAs in human, and 731 protein-coding genes, 39 microRNAs and 78 lncRNAs in mouse (Fig. 1a). These profiles are derived from 1170 different types of cell lines or tissues, the majority of which are MCF-7, HeLa and LNCaP cell lines in human, and liver tissues and V6.5 ES cells in mouse (Fig. 1b). We then performed a systematic transcriptome analysis for each profile, including differential expression analysis, enrichment of Gene Ontology (GO) terms and Kyoto Encyclopedia of Genes and Genomes (KEGG) pathways, extraction of interaction subnetworks, prediction of transcription factor- and microRNA-mediated regulations, identification of cancer/drug associations, and determination of cooperative perturbed genes (e.g., Supplementary Fig. S1). To facilitate the study of context-dependent gene functional mechanisms, we detected (i) novel or cell-specific functions and pathways affected by perturbed genes, (ii) protein interactions and regulatory cascades affected by perturbed genes and (iii) perturbed genes mediating cooperative effects. To make the above resources publicly available and easy to use, we designed a user-friendly web-interface named GPA. We also developed web-based visualization and analysis tools to assist users in identifying correlated perturbed genes based on their own gene lists or transcriptome profiles. The GPA curation procedure is shown in Fig. 2.

### Perturbed genes affecting functions and pathways

**Figure 3:**
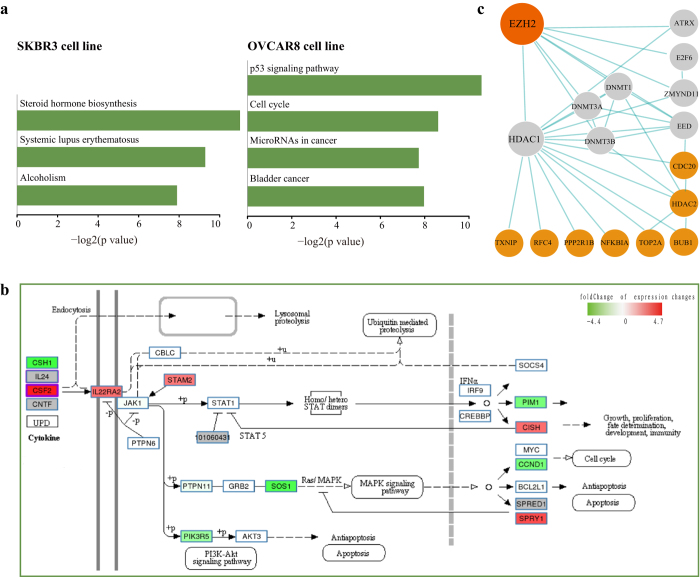
(a) KEGG pathways enriched by DEGs of hsa-miR-1204 perturbation in two different cell lines: SKBR3 and OVCAR8. **(b)** JAK-STAT signaling pathway enriched by BRCA1 knockdown in MCF7. Green indicates decreased gene expression, and red indicates increased gene expression. Highly expressed CSF2 stimulates the JAK-STAT signaling pathway through the tyrosine phosphorylation of STAT3 and, in turn, process downstream signals in the absence of BRCA1. **(c)** Interaction sub-network initiated by EZH2 knockdown in the MCF7 cell line, which implicates EZH2-NFKBIA regulatory cascades mediated by HDAC1. Yellow nodes represent differentially expressed genes caused by EZH2 knockdown.

Gene perturbation is a powerful means to explore gene functions in specific cell contexts because a gene can play distinct roles in different contexts, especially in the case of cell-specific functions or pathways^14^. Therefore, for each perturbed gene, the GPA offers GO functions and KEGG pathways enriched by differentially expressed genes (DEGs) across different cell lines or tissues. We compared perturbation experiments that were generated using the same microarray platform in the same organism for a common gene. We found some cell-specific functions and pathways. For example, hsa-miR-1204 was separately perturbed in the human breast cancer cell line SKBR3 (GPA ID: GPAHSA100105) and in the ovarian carcinoma cell line OVCAR8 (GPA ID: GPAHSA100107), both of which were measured using the Affymetrix HT Human Genome U133A Array. We did not find any common DEGs between these two perturbation experiments (26 DEGs in SKBR3 and 24 DEGs in OVCAR8). Pathway comparisons showed that hsa-miR-1204 could be involved in different pathways in different cell lines, such as ‘Steroid hormone biosynthesis’ in SKBR3 and ‘Cell cycle’ in OVCAR8 (Fig. 3a). As another example, we found a significant overlap (p = 2.05e−8, cumulative hypergeometric test) of DEGs between hsa-miR-221 perturbations in the human breast cancer cell line MCF7-FR (GPA ID: GPAHSA100046) and the prostate cancer cell line PC-3 (GPA ID: GPAHSA100168), both of which were detected using the Affymetrix Human Genome U133 Plus 2.0 Array. We also found different pathways enriched in different cell lines, such as the ‘Chemokine signaling pathway’ in MCF7-FR and the ‘Jak-STAT signaling pathway’ in PC-3 cells. Moreover, GPA enables the discovery of new cellular functions or biological pathways. When investigating functions enriched by DEGs from SOX2 deletion in the human colorectal cancer cell line SW620 (GPA ID: GPAHSA000268), we found novel functions based on comparisons with known functional annotations from GO. Among these functions, two, including the regulation of cell migration and focal adhesion, have been corroborated by a recent study^5^. Some novel functions of SOX2, such as the regulation of viral genome replication and cell-substrate junction, should be further investigated in future studies. Similarly, in the perturbation of PTEN in the human breast cancer cell line MCF-10 A (GPA ID: GPAHSA001288), we found many potential novel functions, such as epithelial cell differentiation, granulocyte migration, epidermis development and wound healing. Among these functions, epithelial cell differentiation has been demonstrated by Qi *et al.* in 2014^15^.

To further understand how perturbed genes induce functional changes in pathways, we provide all enriched KEGG pathway maps and color member genes according to fold changes in expression. For example, we found that DEGs of BRCA1 knockdown in the human breast cancer cell line MCF−7 (GPA ID: GPAHSA000935) were significantly enriched in the JAK-STAT pathway (p < 0.01), indicating that BRCA1 can be involved in the regulation of the JAK-STAT pathway, which is consistent with previous evidence that BRCA1 can constitutively activate JAK-STAT signaling^16^ and participate in the metastasis of breast cancer^17^. As shown in Fig. 3b, BRCA1 knockdown results in remarkable expression change of CSF2, an upstream cytokine in the JAK-STAT signaling pathway, which can stimulate the JAK-STAT signaling pathway through the tyrosine phosphorylation of STAT3 in cancer cells^16^,^18^. Thus, BRCA1 can contribute to the metastasis of breast cancer by influencing the expression of CSF2. Interestingly, we found that BRCA1 knockdown led to the down-regulation of SOS1 and the up-regulation of SPRY1. SOS1 and SPRY1 are the activator and repressor of the Ras/MAPK pathway, respectively^19^,^20^,^21^. The dysregulated SOS1 and SPRY1 induced by BRCA1 knockdown might therefore inactivate the Ras/MAPK pathway, which has an important role in breast cancer.

### Effects of gene perturbation on protein interactions and regulatory cascades

The protein interactions and regulatory cascades affected by perturbed genes are valuable information to explore how dysfunctional information propagates in the protein interaction networks and to infer possible regulatory mechanisms. We provide protein interaction subnetworks centered on perturbed genes and utilize the Cytoscape plugin to visualize the interactions in the GPA. Figure 3c shows the protein interaction subnetwork of EZH2 in the human metastatic breast cancer cell line MDA-MB-231 (GPA ID: GPAHSA000520). Previous studies have confirmed that EZH2 can interact with histone deacetylase 1 (HDAC1) and induce the activity of HDAC1. We find that HDAC1 interacts with numerous DEGs (e.g., NFKBIA) of EZH2 knockdown, suggesting that EZH2 knockdown may affect the activity of HDAC1^22^, which in turn influences the expression levels of these DEGs. Notably, Kleer *et al.* demonstrated that EZH2 participates in cancer cell invasion and breast cancer progression and that EZH2-mediated cell invasion requires histone deacetylase activity^22^. Moreover, previous experiments performed both *in vivo* and *in vitro* strongly suggested the involvement of NF-kappaB in breast cancer^23^. These results implicated EZH2-NFKBIA regulation mediated by HDAC1 in breast cancer. In addition, we provide characterizations of regulatory cascades initiated by perturbed genes through enrichment analysis of transcription factors and microRNAs (Methods). Users can easily and precisely identify transcription factors and/or microRNAs whose target genes are substantially affected by perturbed genes. For example, miR-205 overexpression (GPA ID: GPAHSA100034) caused the down-regulation of transcription factor E2F1 in the LNCaP cell line, and its DEGs were significantly enriched for targets of E2F1 (p < 0.001), indicating that E2F1 may function as a key intermediate element of miR-205-mediated regulatory cascades, which is consistent with previous studies^24^.

### Perturbed genes mediating cooperative effects

Cooperation between genes is crucial for maintaining cell biological processes^25^. Therefore, genes inducing similar transcriptome changes will most likely have similar biological functions or potential cooperation. To identify cooperative effects between different perturbed genes, we calculated the Pearson correlation coefficients of global transcriptome changes between pairs of perturbed genes. For example, the knockdown of lncRNA HOTAIR in the human Primary Foot Fibroblasts (GPA ID: GPAHSA200003) and SUZ12 in the human HepG2 cells (GPA ID: GPAHSA001271) affected many common genes (32%, fold change ≥ 2). We found that their global transcriptome changes showed a statistically significant correlation (p < 0.001, Pearson’s correlation test), indicating that they might function cooperatively. A previous study has shown that HOTAIR can interact with SUZ12 (a core component of Polycomb repressive complex 2 (PRC2)) to promote cancer invasiveness and metastasis^26^, and HOTAIR functions mainly through its 5’ domain binding to PRC2 and in turn altering epigenetic modifications at specific genomic loci^27^. Moreover, the cooperative functions of these genes were found to be involved in many important biological processes of various cancers^26^. Finally, we provide genes possessing underlying cooperative or similar functions for each perturbed gene based on similar transcriptome changes, which can help to characterize gene functions or provide references for perturbation experiments, such as combinatorial knockout.

**Figure 4:**
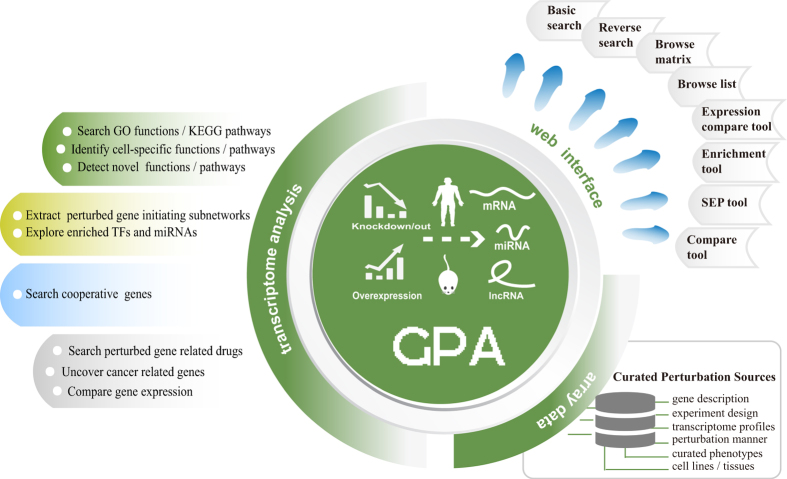
Main applications of the GPA. Applications of GPA transcriptome analysis results mainly aim at detecting (i, green) novel or cell-specific functions and pathways affected by perturbed genes, (ii, yellow) protein interactions and regulatory cascades affected by perturbed genes, (iii, blue) perturbed genes mediating cooperative effects, and (iv, gray) others, mainly focused on cancers and drugs.

In addition, we created Kaplan-Meier survival curves and visualized expression changes of perturbed genes within multiple cancers from TCGA (Supplementary Table 1) in the GPA. To further facilitate the discovery of potential novel cancer-related genes, we measured whether the DEGs of perturbed genes were significantly enriched in multiple cancers using gene set enrichment analysis (GSEA). Moreover, we evaluated the potential therapeutic benefit of perturbed genes by measuring correlations between drugs/small molecules and perturbed genes. We designed several web-based tools for identifying GPA entries associated with user-uploaded gene lists or transcriptome profiles from users’ own experiments. As a whole, the GPA is a valuable resource for the functional genomics community. The GPA contains a large number of curated transcriptome profiles from various perturbation experiments for discovering context-dependent gene functional mechanisms. The GPA provides references for experimental scientists to conduct biomedical experiments. The primary applications of the GPA are shown in Fig. 4.

## Discussion

Over the past decade, increasing numbers of genome-wide transcriptome profiles of gene perturbations have been generated. A large number of studies have performed transcriptome analysis through gene perturbations to explore the underlying molecular mechanisms of the perturbed genes. The GPA is an online database collecting the transcriptome profiles of perturbed genes and providing comprehensive functional characterizations to facilitate the rapid searching, exploration and visualization of gene perturbations along with references for experimental scientists to conduct perturbation experiments.

At present, there are several tools that offer a compendium of gene expression analyses, such as iPathwayGuide ( http://www.advaitabio.com/ipathwayguide.html), CRSD^28^ and CARMAweb^29^, based on user-uploaded gene expression profiles or lists of genes. By comparison, the GPA performs more specific transcriptome analysis under the condition of single-gene perturbations, including the identification of differentially expressed genes, enrichment of GO and KEGG, construction of perturbation networks, prediction of transcription regulation and cancer/drug associations, and assessment of cooperative perturbed genes. Moreover, the GPA database offers useful resources for analyzing genome-wide expression changes after single-gene perturbations. In particular, the GPA can characterize novel or cell-specific functions affected by perturbed genes, provide perturbation networks to explore how dysfunctional information propagates in protein interaction networks, and assess cooperation between perturbed genes.

Currently, the GPA mainly focuses on single-gene perturbations by knockout, RNA interference and overexpression. Most perturbed genes are protein-coding genes. With extensive functional research on non-coding RNAs, non-coding gene perturbation data will quickly accumulate in public resources. We will periodically update the GPA to include these accumulating permutation data on non-coding RNAs, enlarging our database. Multiple genes are generally required to cooperatively contribute to specific phenotypes or biological processes^30^,^31^. Recent studies have revealed that combinatorial perturbations of multiple genes can induce obvious phenotype changes, whereas perturbation of the single genes did not^32^. In the future, the GPA will also include combinatorial gene perturbations to overcome the limitations of single-gene perturbations, such as compensatory effects, genetic buffering, and the redundancy of cellular mechanisms or pathways.

Recently, we observed that large amounts of high-throughput sequencing data were generated from gene perturbations using RNAi, TALEN or CRISPR/CAS systems. These data allow us to capture changes of transcriptome, epigenome and regulatome, providing new insight into the molecular mechanisms of perturbed genes from different layers. For instance, RNA-seq presents the possibility of detecting the expression of non-coding RNAs (e.g., lncRNAs), alternative splicing events and 3` UTR shortening. In next version of the GPA, we also intend to incorporate these data and provide more comprehensive molecular depictions of gene perturbations.

In summary, we will keep the GPA datasets up to date and maintain the GPA as an extensible database. We believe the GPA will become a valuable resource for the functional genomics community and a practical tool for experimental scientists.

## Methods

### Data collection and compilation

We collected transcriptome profiles of single-gene perturbations across different cell types or tissues in human and mouse by searching all experimental datasets from GEO using the keywords ‘knock out’, ‘knock down’, ‘RNAi’, ‘knock in’, ‘overexpression’, ‘high expression’, ‘low expression’, ‘siRNA’ or ‘shRNA’. For each collected profile, we extracted the samples in which a specific gene is perturbed as case samples. The samples described with keywords of ‘scramble’, ‘non-specific’, ‘empty control’, ‘negative control’, ‘non-targeting control’, ‘untreated control’, ‘ethanol control’ or ‘wild-type’ in the sample description of GEO were defined as control samples. Other experimental information, such as perturbed gene symbol, cell line, microarray platform, perturbation manner and experiment design, was also compiled with the corresponding profile. This process was triple-checked by different researchers. For the convenience of downstream analysis, the probe identifiers of each transcriptome profile were converted to Entrez Gene IDs.

### Database architecture and web interface

The GPA is built on Apache Tomcat 7.0.47 with MySQL 5.6.14 (database server) at the back end and HTML, JSP 2.1 and JavaScript at the front end to provide a user-friendly interface for locating and retrieving information from the database. Apache, MySQL, and JSP are preferred because they are open-source software and platform independent. The architecture of the GPA database is shown in Fig. 2.

### Organization of data

#### Primary data

Expression profiles of gene perturbations together with the corresponding information, including permutation manner, number of differential genes, experimental design and out-links to other databases, were compiled as the primary data. For each profile, a unique GPA ID was assigned (e.g., GPAHSA000001), which represented a form of global address in the database.

#### Secondary data

Secondary data were derived from various transcriptome analyses for each collected primary profile. These data mainly include DEGs, enriched GO terms and KEGG pathways, interaction subnetworks, enriched transcription factors and microRNAs, correlated cancers and drugs, and correlations between different perturbed genes. These secondary data will assist experimental scientists in further harnessing the power of these profiles, focusing on the detection of (i) novel or cell-specific functions and pathways affected by perturbed genes, (ii) protein interactions and regulatory cascades affected by perturbed genes and (iii) perturbed genes mediating cooperative effects for each collected transcriptome profile.

### Transcriptome analysis of gene perturbations

#### Differential expression analysis

For each transcriptome profile, we used the t test to calculate p values with FDR correction and computed the fold changes of genes by comparing perturbed samples and their corresponding controls. Finally, the genes with fold change ≥2 were identified as DEGs.

#### Cumulative hypergeometric test for enrichment analysis

Enrichment analysis of DEGs was performed mainly based on the cumulative hypergeometric test. The statistical significance of the enrichment was calculated using the following formula (1):


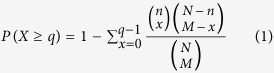


where N is the total number of genes in the transcriptome profile, M is the number of DEGs, n is the number of genes annotated in a certain functional category and q is the number of overlapping genes between DEGs and the functional category.

#### GO and KEGG pathway enrichment analysis

GO terms for the Entrez genes were downloaded from the NCBI ftp site. The KEGG pathways were downloaded from the KEGG database. For each transcriptome profile, the significance of enrichment analysis of DEGs with GO terms and pathways was determined using a hypergeometric test with FDR correction (FDR ≤ 0.05).

#### Evaluating new GO functions and KEGG pathways of perturbed genes

A GO term (or KEGG pathway) enriched by DEGs of the perturbed gene but not annotated by the perturbed gene was considered as a new function (or pathway).

#### Extracting interaction subnetworks

Protein interaction networks were obtained from the Mentha Database^33^ on June 2014, containing 141,247 interactions in human and 22,213 in mouse. For each perturbed gene, its directly interacting genes and DEGs at a distance of two steps in the protein interaction network were extracted to construct its initiated subnetwork.

#### Transcription factors and microRNA enrichment analysis

Transcription factors and their target genes were downloaded from ChIPBase^34^. We obtained two million transcription factor-gene regulatory relationships involving 120 and 69 transcription factors for human and mouse, respectively. In addition, 2207 microRNAs and 76,303 microRNA-target relations in human were retrieved from TargetScan^35^ with a context + score ≤ −0.3. Enrichment analysis of transcription factors and microRNAs was performed by comparing the DEGs of perturbed genes to target genes of transcription factors or microRNAs using a hypergeometric test (FDR ≤ 0.05).

#### Enrichment analysis of perturbed genes with multiple cancers and drugs

Gene expression profiles of thirteen types of cancers were downloaded from TCGA (Supplementary Table 1), which contained 4691 cancer samples and 422 normal samples. Gene set enrichment analysis (GSEA) was performed to measure whether the DEGs of specific perturbed genes showed significant expression changes in multiple cancers (FDR ≤ 0.25). Individual drugs and their target genes were derived from DrugBank^36^. Active compounds and drug-induced gene expression profiles were downloaded from Connectivity Map^37^ (cmap). Finally, 10,399 drug-target relationships from DrugBank and more than 7000 expression profiles from cmap were taken for enrichment analysis of perturbed genes with drugs by a hypergeometric test (FDR ≤ 0.05).

#### Correlation analysis among gene perturbation*s*

For each perturbed gene in GPA, other perturbed genes sharing similar patterns of global expression changes were identified as cooperative genes or genes with similar functions. The Pearson correlation test was used to identify significantly correlated perturbed genes (p ≤ 0.05 and Pearson correlation coefficient ≥ 0.5).

### Implementation of web-based tools

#### Data searching

The GPA provides three search modules with which to extract transcriptome profiles and the corresponding analysis results. In the simple search module, a search box enables users to retrieve data based on gene name (e.g., PTEN, has-miR-100 and HOTAIR) or GPA id (e.g., GPAHSA000001). The advanced search module provides a more detailed search for perturbation data by organism, gene type, perturbation manner and cell line. To facilitate a user-friendly text search, we adopted the jQuery AutoComplete technique to guide users for keyword selection ( http://jqueryui.com/autocomplete/). We also designed a reverse search module to help users retrieve perturbation data by searching their secondary data, such as DEGs, enriched GO terms, enriched KEGG pathways and correlated drugs.

#### Data browsing

We designed two browsing facilities (matrix view and list view) to provide an overall view of gene perturbations in the GPA. Users can search and sort all entries in the GPA by setting various options: (i) organism (*Homo sapiens* and *Mus musculus*), (ii) gene names, (iii) cell lines or tissues, and (iv) gene types (protein-coding gene, lncRNA and microRNA). In the matrix view, users can also flexibly choose multiple perturbation data by clicking green blocks within the browsing matrix table and seek details by clicking the ‘build archive’ button.

#### Gene list enrichment tool

The gene list enrichment tool was designed for retrieving the perturbation data whose DEGs were enriched for the user-uploaded list of gene IDs or symbols by the cumulative hypergeometric test. Users can obtain enriched GPA entries to help characterize the custom gene list.

#### Similar Expression Pattern (SEP) tool

The SEP tool was developed for exploring similar changes in gene expression between perturbation datasets and user-uploaded profiles. This tool utilizes the Pearson correlation test to obtain correlated perturbation entries from the GPA for each user-uploaded profile by measuring the correlations of their log2-fold changes.

#### GPA comparison tool

The GPA comparison tool is used to compare any two GPA datasets to find similarities and differences in the differentially expressed genes, biological functions and pathways, transcription regulators, cancer/drug associations and cooperative perturbed genes. Before comparison, the GPA assesses the comparability between the two datasets by comparing additional information, including their organisms, experimental platforms, cells or cell lines, perturbed genes and perturbation manners. By default, the same organism and experimental platform are required when comparing two perturbation experiments.

#### Download

The download module supports both single and batch download. The single download can be used to extract perturbed data by GPA id (e.g., GPAHSA000001) in our database, and the batch download lists all perturbed differential expression profiles categorized by protein-coding gene, lncRNA and microRNA in human and mouse.

#### Updating of GPA

We will incorporate newly released data every month. The GPA also allows users to submit custom perturbation data. However, before including custom data in the GPA, our team will scrutinize the authentication of the data.

## Additional Information

**How to cite this article**: Xiao, Y. *et al.* Gene Perturbation Atlas (GPA): a single-gene perturbation repository for characterizing functional mechanisms of coding and non-coding genes. *Sci. Rep.* **5**, 10889; doi: 10.1038/srep10889 (2015).

## Supplementary Material

Supplementary Information
