# Supplementary material for: Gene Perturbation Atlas (GPA): a single-gene perturbation repository for characterizing functional mechanisms of coding and non-coding genes
Source: Sci Rep. 2015 Jun 3;5:10889. doi: 10.1038/srep10889 (PMC4650632; doi:10.1038/srep10889)
Supplement: Supplementary Information [file srep10889-s1.pdf]

Gene Perturbation Atlas (GPA): a single-gene perturbation repository for characterizing functional mechanisms of coding and non-coding genes

Yun Xiao[1,2,+], Yonghui Gong[1,+], Yanling Lv[1,+], Yujia Lan[1,+], Jing Hu[1], Feng Li[1], Jinyuan Xu[1], Jing Bai[1], Yulan Deng[1], Ling Liu[1], Guanxiong Zhang[1], Fulong Yu[1], Xia Li[1,*]

[1]College of Bioinformatics Science and Technology, Harbin Medical University, Harbin, Heilongjiang 150086, China.
[2]Key Laboratory of Cardiovascular Medicine Research, Harbin Medical University, Ministry of Education.

[+]These authors contributed equally to this work.
*Correspondence should be addressed to Xia Li (lixia@hrbmu.edu.cn)

Supplementary Table S1: Multiple cancers from TCGA.

Cancer	Name	Cancer samples	Normal samples
BRCA	Breast invasive carcinoma	526	64
COAD	Colon adenocarcinoma	155	19
OV	Ovarian serous cystadenocarcinoma	541	18
GBM	Glioblastoma multiforme	528	10
BLCA	Bladder Urothelial Carcinoma	241	19
CESC	Cervical squamous cell carcinoma and endocervical adenocarcinoma	185	3
HNSC	Head and Neck squamous cell carcinoma	495	43
PRAD	Prostate adenocarcinoma	297	50
READ	Rectum adenocarcinoma	69	3
KIRC	Kidney renal clear cell carcinoma	518	72
LUAD	Lung adenocarcinoma	488	58
UCEC	Uterine Corpus Endometrial Carcinoma	158	13
LUSC	Lung squamous cell carcinoma	490	50
Total		4691	422

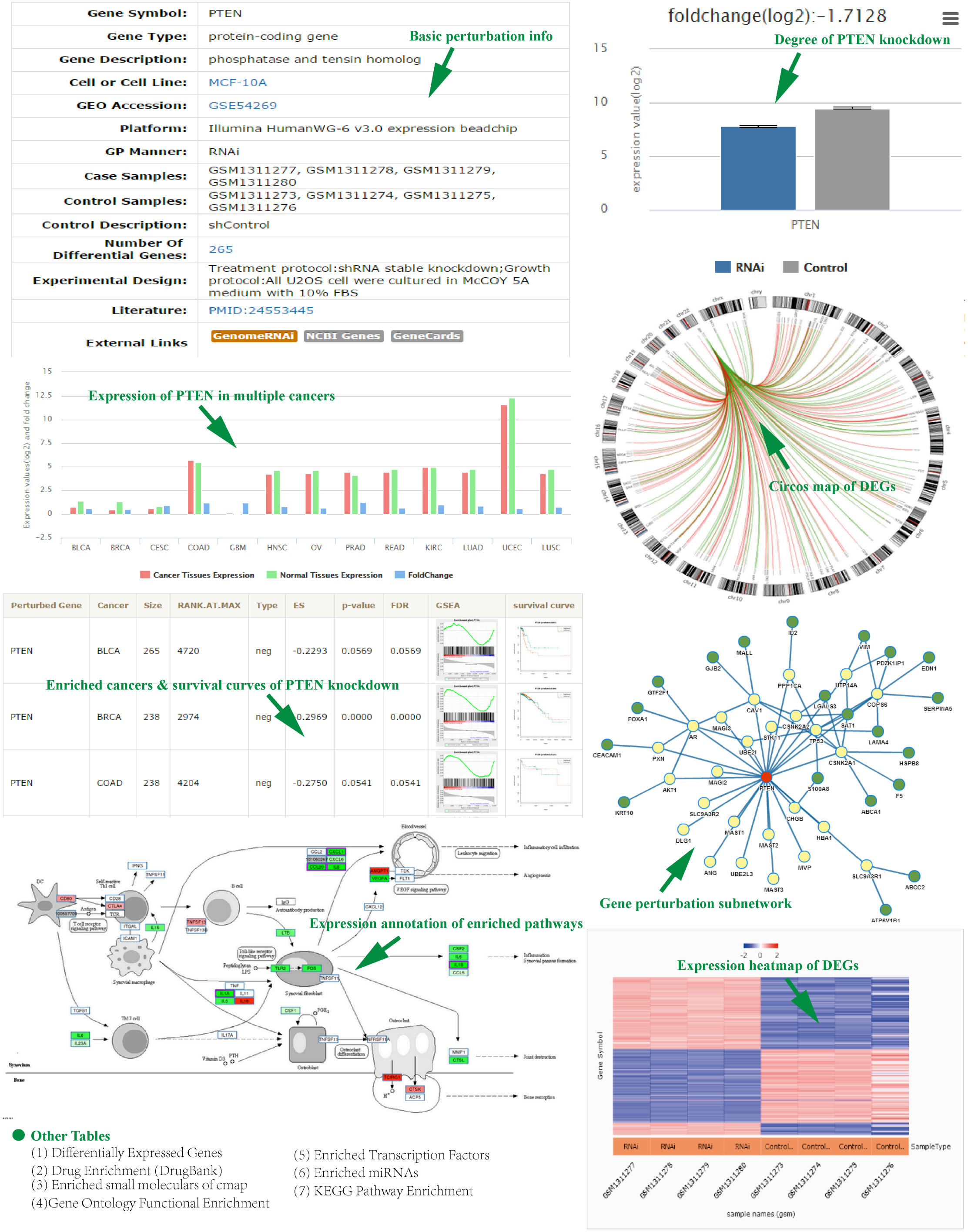

Supplementary Figure S1: The transcriptome analysis results of PTEN knockdown.
